# Pteropine Orthoreovirus in an Angolan Soft-Furred Fruit Bat (*Lissonycteris angolensis*) in Uganda Dramatically Expands the Global Distribution of an Emerging Bat-Borne Respiratory Virus

**DOI:** 10.3390/v12070740

**Published:** 2020-07-09

**Authors:** Andrew J. Bennett, Tony L. Goldberg

**Affiliations:** 1 Department of Pathobiological Sciences, University of Wisconsin-Madison, Madison, WI 53706, USA; abennett3@wisc.edu; 2 Global Health Institute, University of Wisconsin-Madison, Madison, WI 53706, USA

**Keywords:** *pteropine orthoreovirus*, bats, *reoviridae*, emerging respiratory infections

## Abstract

Pteropine orthoreovirus (PRV; *Reoviridae*: *Spinareovirinae*) is an emerging bat-borne zoonotic virus that causes influenza-like illness (ILI). PRV has thus far been found only in Australia and Asia, where diverse old-world fruit bats (*Pteropodidae*) serve as hosts. In this study, we report the discovery of PRV in Africa, in an Angolan soft-furred fruit bat (*Lissonycteris angolensis ruwenzorii*) from Bundibugyo District, Uganda. Metagenomic characterization of a rectal swab yielded 10 dsRNA genome segments, revealing this virus to cluster within the known diversity of PRV variants detected in bats and humans in Southeast Asia. Phylogeographic analyses revealed a correlation between geographic distance and genetic divergence of PRVs globally, which suggests a geographic continuum of PRV diversity spanning Southeast Asia to sub-Saharan Africa. The discovery of PRV in an African bat dramatically expands the geographic range of this zoonotic virus and warrants further surveillance for PRVs outside of Southeast Asia.

## 1. Introduction

Pteropine orthoreovirus (PRV; *Reoviridae*: *Spinareovirinae*) is a bat-borne zoonosis that causes upper respiratory tract infection (URTI), ranging from mild respiratory symptoms to influenza-like illness (ILI), in Southeast Asia [1,2,3,4]. PRV is a fusogenic orthoreovirus with 10 dsRNA segments (S1-4, M1-3, L1-3) [5]. PRV is only distantly related to other mammalian orthoreoviruses (MRV), and is nested within a clade of fusogenic viruses that cause significant pathology in reptiles and birds [5]. First isolated in 1968 from a flying fox (*Pteropus poliocephalus*) in Australia [6], variants of PRV have since been detected in diverse pteropodid bats across Asia [7,8,9,10] and in residents of, and travelers to, Southeast Asia [1,11,12,13]. Here, we describe the discovery of PRV in a bat from Equatorial Africa and discuss the implications of a dramatically expanded PRV geographic distribution.

The reoviruses (respiratory enteric orphan viruses; family *Reoviridae*) contain non-enveloped, segmented dsRNA viruses that cause distinct pathologies in diverse hosts [14]. The *orthoreovirus* genus is split into fusogenic and nonfusogenic viruses, determined by their capacity to form multinucleated syncytia mediated by uniquely simple fusion-associated small transmembrane (FAST) proteins for which there are no known homologs elsewhere in nature [15]. For nearly 30 years, the original variant of PRV, Nelson Bay virus (PRV1NB), remained a singular curiosity as the lone mammalian fusogenic orthoreovirus [16].

Since 1999, 15 PRVs (Table 1) were discovered as infecting pteropid bats in China, Indonesia, Malaysia, and the Philippines [7,8,9,10,17], and direct evidence of spillover to humans has been reported in Malaysia and Indonesia [1,11,12,13,18,19]. In 2006, Melaka virus (PRV3M), and shortly thereafter Kampar virus (PRV4K), were the first PRVs isolated from patients in Malaysia presenting to clinics with high fever, acute respiratory illness, vomiting, and diarrhea [18,19]. In each instance, the index case had had recent but indirect contact with bats, and in each instance human-to-human transmission occurred [18,19]. Subsequently, acute human PRV infections were identified in travelers who had visited caves in Bali, Indonesia [11,12]; in a Malaysian man with no clear bat exposure [20]; and in 34 out of 200 patients presenting with URTI to a Malaysian health clinic in the summer of 2012 [1]. Serological surveys suggest that zoonotic PRV infections may be common in Southeast Asia. For example, a survey of people in Tioman Island, Malaysia, detected antibodies in 13% of residents (*n* = 109; virus neutralization test) [18], and a survey of 272 hospital patients in Vietnam found that 4% had anti-PRV IgG antibodies by ELISA [21].

In June and July of 2017, we sampled Angolan soft-furred fruit bats (*Lissonycteris angolensis ruwenzorii*) in Kasitu Subcounty, Bundibugyo District, Western Region, Uganda, which was the site of a 2007–2008 outbreak of ebolavirus disease [22]. We sampled bats from a culvert beneath a road spanning a mountain stream that serves as a water source for nearby human communities. We then used metagenomic methods to survey for viruses that may have been shed into the stream. In one rectal swab, we identified the nearly complete genome of the first African bat PRV, which we provisionally named Kasama virus (PRV16K) (named for Kasitu Subcounty, Uganda, and the Konjo word for water, *amaghetse*). 

The genome sequence of PRV16K is highly similar to that of Asian PRV variants and PRV16K clusters within the known diversity of Asian PRVs, despite its geographic distance from Asia (see below). In Southeast Asia, PRV variants appear to transmit readily between species of pteropid bats [23]. We hypothesize that this promiscuity could result in a multispecies geographic continuum of PRV diversity in pteropid bats from Southeast Asia to Equatorial Africa. In support of this hypothesis, we present evidence of isolation-by-distance (IBD) for PRV. Given the apparent frequency of PRV transmission from bats to humans in Southeast Asia, PRV may represent an unappreciated cause of ILI across Africa and Asia.

## 2. Materials and Methods 

### 2.1. Collection of Samples

The bats were collected by mist-net (Avinet, Portland, ME, USA) as they exited their roost at dusk. The bats were roosting as a multispecies assemblage of ~200 Angolan soft-furred fruit bats (2 nights of double-observer roost exit counts) and an unknown number of Sundevall’s leaf-nosed bats (*Hipposideros caffer*). The roost was a stone and cement culvert beneath the Itojo-Sempaya Old Road in Bundibugyo District, Uganda (0°50’18.5”N and 30°10’22.1”E). The bats were held in individual cloth drawstring bags from the time of collection until sampling, they were provisioned with a sugar/water solution (30% m/v) following sample collection, and they were released on site. 

Rectal swabs were collected from 11 Angolan soft-furred fruit bats using sterile rayon-polyester-tipped swabs, preserved in TRI Reagent (Zymo Research, Irvine, CA, USA), and frozen at −20 °C within 3 h of collection. All the samples were exported with permission of the Uganda Wildlife Authority and the Uganda National Council for Science and Technology, shipped in accordance with International Air Transport Association (IATA) regulations, and imported under permit number 2017-07-103 of the Centers for Disease Control and Prevention (Atlanta, GA, USA).

### 2.2. Metagenomics

Total RNA was extracted from the bat rectal swab samples using the Direct-zol RNA MicroPrep kit (Zymo Research, Irvine, CA, USA). Libraries were prepared for high-throughput sequencing (HTS) as previously described [24]. Briefly, sequencing libraries were prepared from 1 ng of cDNA per sample using the Nextera XT DNA (Illumina, San Diego, CA, USA) sample kit and sequenced using the Illumina MiSeq platform (Illumina, San Diego, CA, USA). HTS reads were quality-trimmed, and contiguous sequences (contigs) were assembled de novo using CLC Genomics Workbench version 11.1 (CLC Bio, Aarhus, Denmark) as previously described [25]. The assembled contigs were compared at the nucleotide and deduced protein sequence level to virus-derived and bat mitochondrial gene sequence in GenBank using BLASTn and BLASTx homology searching algorithms, respectively [26]. 

### 2.3. Phylogenetics of PRV16K Segments and Asia Pacific PRVs 

PRV has 10 dsRNA genome segments (S1-3, M1-3, L1-3), nine of which contain a single open reading frame (ORF) (S2-3, M1-3, L1-3) and one (S1) with 3 overlapping ORFs [27]. The ORFs of the 10 segments of PRV16K were aligned (GenBank Accession numbers MT505315–MT505324) with homologous ORFs of the 15 previously identified strains of PRV [28] (Table 1; Genbank Accession numbers in Supplemental Table S1) using the Prank algorithm implemented in TranslatorX. The phylogenies were inferred using PhyML with “Bayesian Information Criterion” (BIC) smart model selection, “general-time reversible model with incorporation of rate of variation across site and proportion of invariable sites” (GTR+Γ+I) [29] and these are visualized in FigTree v.1.4.3 with midpoint rooting. The pairwise p-distances and standard errors (estimated from 1000 bootstraps) were calculated for all available PRV segments using MEGA7 [30].

### 2.4. Detection of Positive Selection on Cell-Attachment Protein (σC)

Sixteen sequences (PRV1NB–PRV16K; Table 1) of the S1 ORF encoding the cell-attachment protein (σC) were aligned as previously described [31]. The ratio of nonsynonymous (Ka) to synonymous (Ks) nucleotide substitution rates was estimated and a Ka/Ks annotated tree was generated using the Ka/Ks Calculation Tool of the University of Bergen Computational Biology Unit [32].

### 2.5. Correlation of Geographic Distance with Patristic Distance of Segments S1-4 

To examine the patterns of the geographic spread of PRVs, we measured the pairwise geographic distance (minimizing distance traveled over water) between the site of sample collection/reported sites of origin for 15 PRVs (excluding PRV12I, which was isolated from a bat imported to Italy) (Supplemental Table S1) using ArcGIS Online (ESRI, Redlands, CA, USA). We computed the patristic distances between PRV variants from the phylogenetic trees of segments S1-S4 using PATRISTIC [33]. Mantel tests of the matrix correlation [34] were then performed to compare matrices of pairwise geographic distance to matrices of pairwise patristic distance for S1-S4 using XLSTAT [35]. The confidence intervals and the significance of Mantel’s r (rM) were computed using 10,000 Monte Carlo simulations (alpha = 0.05) and Mantel correlograms were generated using the Spatial Data Calculator [36] (20 intervals of 1000 km).

## 3. Results

### 3.1. Sequencing and PRV Genome Assembly

We collected rectal swabs from 11 bats, representing an estimated 5.5% of the approximately 200 Angolan soft-furred fruit bats in the roost. Metagenomic sequencing revealed the presence of PRV in one of these bats (prevalence 9.1%). In the infected bat, the average depth of coverage across the 10 PRV segments was 82.15 ± 32.7, with 1.2% of quality-trimmed reads mapping to a PRV segment. Gaps were filled via rt-PCR and Sanger sequencing. The coding-complete sequence of 9 out of 10 PRV segments was recovered. Based on the sequence data, PRV16K has the typical PRV genome organization including a tricistronic S1 and single ORFs on the remaining segments. For L3, 706 nt of the ORF encoding the major inner capsid protein (λ1) (nt 2544–3249 of L1) was lost to low coverage and could not be recovered by rt-PCR. This gap in the sequence alignment of L3 was excluded for all subsequent analyses. The mitochondrial gene sequences of cytochrome b (*cytb*) and cytochrome c oxidase subunit 1 (*cox1*) from the infected bat were also assembled as described above. These sequences (GenBank accession numbers MT708578 and MT707261) confirmed the bat to be an Angolan soft-furred bat of the *Ruwenzorii* subspecies (*Lissonycteris angolensis ruwenzorii*) (percent identity: *cytb* 99.56; *cox1* 100%). 

### 3.2. Evolutionary Relationships among PRV Segments 

**Figure 1:**
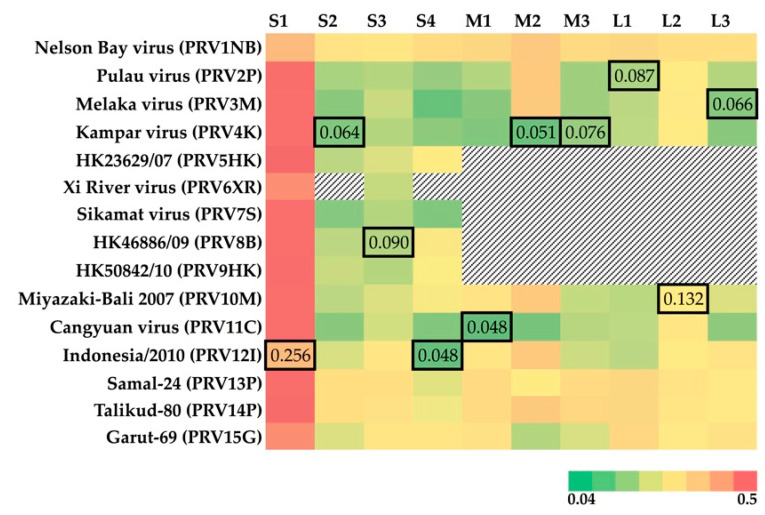
Heatmap of p-distance for the segments of 15 previously isolated PRVs relative to PRV16K. The pairwise p-distance was estimated using MEGA7 (1000 bootstrap replicates). The GenBank accession numbers for PRV isolates are available in Supplemental Table S1. The missing segments of PRV isolates are indicated by black and white hashed cells. The outlined cells indicate the lowest p-distance to PRV16K for each segment. The heatmap color key shows the range of the p-distance values estimated.

**Figure 2:**
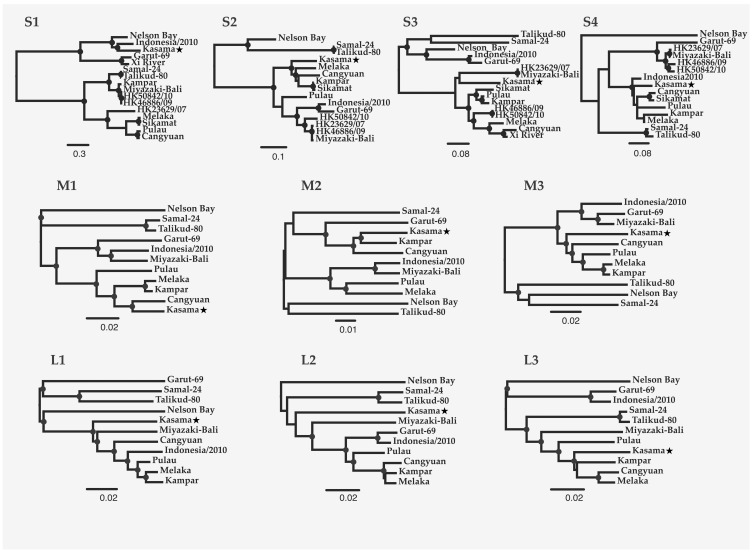
Maximum likelihood trees of the 10 PRV genome segments. The trees were constructed from codon-based alignments of open reading frames from each segment (GenBank Accession numbers in Supplemental Table S1), with molecular evolution model selection (GTR+ +I) by PhyML smart model selection (BIC). Circles on nodes indicate >75% confidence based on 1000 bootstrap replicates. The scale bar indicates nucleotide substitutions per site.

Nucleotide-level analyses revealed different patterns for the 10 PRV segments (Figure 1). PRV S1 demonstrates the greatest diversity globally with an average pairwise distance of 35% ± 12.7%. The tricistronic S1 segment encodes the cell-attachment protein (σC), as well as two nonstructural proteins (p10: fusion-associated small transmembrane (FAST) protein, and p17 (unknown function in PRV)) [37]. Much of the global PRV S1 nucleotide diversity is found in the σC ORF (overall mean p-distance 40.6% ± 0.7%) as compared to P10 (24.1.x% ± 1.4%) and P17 (30.4% ±1.5%). PRV16K form a clade with bat variants PRV1NB, PRV12I, and PRV15G, but clusters with different human and bat variants across the remaining segments (Figure 2).

PRV16K S1 is most closely related to S1 of PRV12I, from a bat imported to Italy from Indonesia [9] (p-distance = 25.3% ± 1.1%). For the remaining segments, p-distance is remarkably low between PRV16K and known variants from humans (PRV3M, L3 (6.6% ± 0.5%); PRV4k, S2 (6.4% ± 0.4%), M2 (5.3% ± 0.5%), M3 (7.6% ± 0.5%); PRV8B, S3 (9.0% ± 0.6%); PRV10M, L2 (13.2% ± 0.6%)) and from bats (PRV2P, L1 (8.7% ± 0.5%); PRV11C, M1 (4.8% ± 0.4%); PRV12I, S4 (4.8% ± 0.4%)) of Southeast Asia (Figure 1). 

### 3.3. Geographic Relationships among PRV Segments S1-4

**Figure 3:**
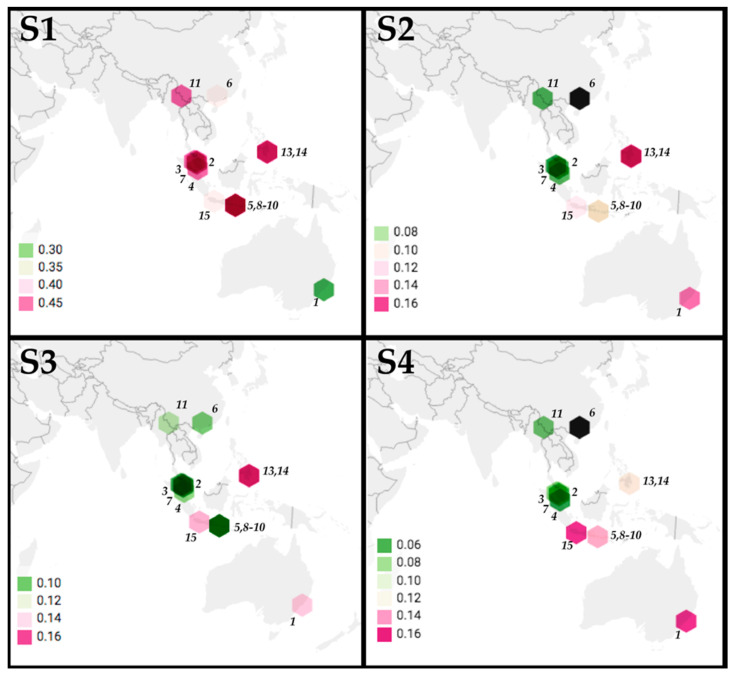
Phylogeographic analysis of PRV segments S1-S4. The p-distances between PRV16K segments S1-4 and segments S1-S4 of 14 PRV isolates (MEGA7, 1000 bootstrap replicates) are indicated by hexagons indicating the locations of origin of PRV isolates, with hexagons colored to indicate p-distance values (scale bars indicate ranges for each segment). The numbers indicate PRV isolate: (1) PRV1NB, (2) PRV2P, (3) PRV3M, (4) PRV4K, (5) PRV5HK, (6) PRV6XR, (7) PRV7S, (8) PRV8B, (9) PRV9HK, (10) PRV10M, (11) PRV11C, (12) PRV12I, (13) PRV13P, (14) PRV14P, (15) PRV15G. The GenBank accession numbers are in Supplemental Table S1. The sequences for S2 and S4 were not available for (6) PRV6XR (indicated by a black hexagon).

**Figure 4:**
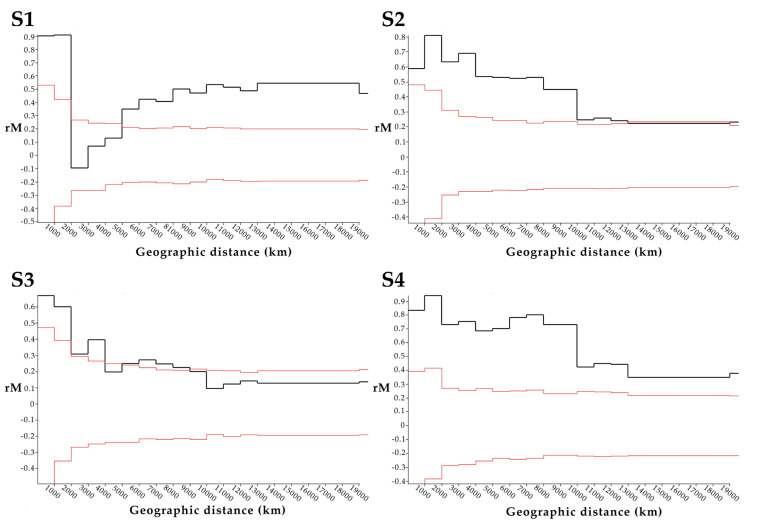
Mantel correlograms for segments S1-4 of 16 PRV isolates. The mantel correlograms show the relationship between geographic distance (km) and the correlation between pairwise genetic and geographic distances (Mantel’s r, or rM) across 20 distance intervals (1000 km). The black lines show Mantel’s r computed for each interval, while the red lines show the results of 10,000 random permutations (randomizing matrix associations). For each interval, Mantel’s r values (black lines) outside of the limits of the results of 10,000 random permutations (red lines) are considered significant (alpha = 0.5).

The location of origin of 14 PRV variants were mapped with the color of points indicating p-distance relative to PRV16K of S1-4 (Figure 3). Although p-distances generally increase with geographic distance for S1-S4, the low S1 pairwise distance between PRV16K and PRV1NB is an outlier for the segment. The global Mantel test of the correlation between geographic and patristic distance of PRV S1-4 indicated significant correlation for S1, S2, and S4 but not for S3, and correlation was strongest for S1 and S4 (Table 2). The mantel correlograms (Figure 4) show some distance decay of Mantel’s r (rM), but genetic distance significantly declines with geographic distance out to the greatest pairwise geographic distance (19,420 km) for S2 and S4. The correlogram for S1 shows a a steep decline in rM from 2000 to 5000 km, which suggests that forces other than geographic distance, such as diversifying selection, may be driving geographic clustering of S1 genetic diversity. The global Mantel test for S3 was not significant, but the mantel correlogram for S3 shows significant correlation at short and intermediate distance intervals (0–4000 km, 5000–9000 km; Figure 4).

### 3.4. Diversifying Selection on Cell-Attachment Protein (σC) 

We detected strong positive selection (Ka/Ks = 1.231) at node 15, where the two major clades of PRV S1 diverged. The purifying selection predominates (Ka/Ks << 1) on all other branches aside from the more moderate positive selection (Ka/Ks = 1.007) on branch 2 of Node 7 (Supplementary Figure S1).

## 4. Discussion

*PRV* was first discovered in 1968, with the isolation of PRV1NB from a grey-headed flying fox (*Pteropus poliocephalus*) [6]. PRV1NB was a novelty among mammalian orthoreoviruses, as it was capable of forming multinucleated syncytia via a FAST protein akin to the avian reoviruses (ARV) [5]. PRV2P, isolated from a small flying fox (*Pteropus hypomelanus*) in Malaysia in 1999, expanded the range of PRV in bats [10], but the recognition of PRV as a cause of respiratory illness in humans did not come until the discovery of PRV3M and PRV4K associated with URTI in Malaysia [18,19]. This realization led to further investigations showing that PRV may be transmitted regularly from bats to humans [1,20], placing it in a rare class of zoonotic bat viruses that transmit frequently from bats to humans [38]. The discovery of a closely related PRV strain in a Ugandan bat expands the geographic distribution of the virus to a new continent. The high similarity of PRV16K to Southeast Asian variants, and the strong and significant correlation between the geographic and genetic distances of PRV segments, evince that PRV16K is not the extreme outlier its far-flung origin would suggest, but perhaps a distant point on a geographic continuum of PRV diversity.

PRV16K, discovered in a rectal swab from an apparently healthy Angolan soft-furred fruit bat in Bundibugyo District, Uganda, is closely related to PRVs isolated from humans and bats in Asia Pacific (Figure 1). The ORFs of 8 out of 10 segments (S2-4, M1-3, L1, L2) all share ≥91% nucleotide identity with a known PRV variant. S1 identity is lower, but globally S1 appears to be less constrained than the remaining segments across PRVs. That no segments of PRV16K appear as an outlier to an Asian clade of PRVs (Figure 2) suggests that the virus did not co-diverge with African fruit bats from their ancestral Asian lineages in the Miocene [39], but rather spread between African and Asian bats more recently. The Angolan soft-furred bats are split into five subspecies. PRV16K was identified in the *Ruwenzorii* subspecies, which has been identified across sub-Saharan Africa [40].

The genetic and geographic distances between PRVs correlated significantly for segments S1, S2, and S4 by the global Mantel test (Table 2). Significant Mantel correlation supports an isolation-by-distance (IBD) hypothesis [41], wherein genetic distance between PRV variants increases with geographic distance across a distribution of host species [42]. For S2 and S4, Mantel correlograms (Figure 4) showed a relatively smooth decrease in rM, with significant correlation out to the greatest distance interval. This provides evidence that S2 and S4 diversity, across the geographic distribution of PRV, is not significantly structured by factors such as host species. In other words, PRV appears to have a broad host tropism [3]. We note that our analyses assume that human PRV infections were acquired locally, or, for imported cases, at the reported site of infection. Furthermore, PRV in bats may also be oversampled at some sites, which could cause other geographic biases. The strong association between PRV genetic and geographic distance that we documented may therefore change in magnitude as new viral variants are described from other locations.

Globally, PRV segments show different evolutionary histories (Figure 3), probably due to frequent inter-species transmission and genome reassortment during PRV co-infection [23]. A study of bat roosts in the Philippines found evidence of cross-species transmission of PRV isolates, neutralizing antibodies in the sera of infected and uninfected bats, and one bat apparently co-infected with two genetically divergent PRV isolates [7]. PRV has thus far been isolated from cave- and canopy-roosting frugivorous Pteropodid bats [43]. Cave-roosting bats show a higher propensity for interspecies virus transmission than do canopy roosting bats, due to the co-roosting of multiple species [44]. Although the mechanism by which PRV transmits between bat species across the range of the virus remains incompletely known, these studies suggested that co-roosting behavior is a major driver of such transmission. The transmission of PRV between related bat species should occur with relative ease under permissive ecological conditions, given the aforementioned zoonotic potential of PRV and further evidence of infection of wild crab-eating macaques (*Macaca fascicularis*) in Singapore [3] and Thailand [45]. We anticipate that future surveys of pteropids bats of south Asia, the Middle East, and the Horn of Africa may further expand the range of PRV and fill the gaps in the geographic continuum of genetic distance that we have documented. 

The S1 Mantel correlogram (Figure 4) shows some evidence of genetic clustering, due to the steep decline in rM in the intervals from 2000 to 5000 km. We hypothesized that diversifying selection may have significantly impacted the evolutionary history of S1. We found evidence of strong positive selection on σC, and in particular the ratio of nonsynonymous to synonymous mutations showed strong positive selection acting on the node separating the two S1 clades (Supplemental Figure S1). σC is necessary for cell attachment and may determine cell/host tropism, although the host ligand(s) for PRV σC is not certain [37]. The structural and functional homolog in the more thoroughly studied ARV (σ1) induces high levels of strain specific anti-ARV neutralizing antibodies in birds [46], and so the strong positive selection on σC which orders the evolutionary history of S1 in PRV may be due to immune selection. 

Although we do not yet have evidence that PRV16K is zoonotic, the high similarity of many PRV16K segments to PRVs isolated from humans with ILI suggests that further investigation is warranted. PRV infection dynamics are poorly understood, and the modes of transmission between bats, and to spillover hosts, are largely unknown, but the detection of virus RNA in bat excreta indicates that fecal–oral transmission could play a role. That the bat population in which PRV16K was discovered was associated with a human water source in Bundibugyo District, Uganda, is of concern. Reoviruses are notably stable in the environment due to their non-enveloped, “double-shelled” capsid [47]. Although waterborne transmission of PRV has not been reported, infectious mammalian reoviruses are frequently isolated from surface water [48,49]. Additionally, the original zoonotic variants of PRV (PRV3M, PRV4K) were acquired via indirect contact with bats [18,19], implicating environmental transmission of the viruses. We also note that Bundibugyo District was the site of an ebolavirus disease outbreak in 2007–2008 [22], suggesting a high risk for bat-associated zoonoses in this area.

Surveillance for influenza and ILI in many sub-Saharan African countries is poor, although the disease burden of ILI is significant [50,51,52,53]. Even when active influenza surveillance is undertaken, differential diagnosis requires multiplex diagnostics to accurately identify the diverse etiologies of ILI [54]. It is therefore entirely conceivable that PRV is a cause of URTI in Africa that has gone undetected. Furthermore, given the pan-equatorial African distribution of the Angolan soft-furred fruit bat and the apparent ease of transmission of PRV between bat species reported in Southeast Asia, PRV may be broadly distributed across the African continent. The discovery of PRV in Africa dramatically expands the range of this bat-borne zoonosis and warrants further investigation to assess whether PRV is actively transmitted in sub-Saharan Africa, as it is in Southeast Asia. 

## Figures and Tables

**Table 1:** Pteropine orthoreoviruses detected in bats and humans.

Isolate	Common Name	Year	Host	Country of Origin
PRV1NB	Nelson Bay virus	1968	Bat (*Pteropus policephalus*)	Australia
PRV2P	Pulau virus	1999	Bat (*Pteropus hypomelanus*)	Malaysia
PRV3M	Melaka virus	2006	Human	Malaysia
PRV4K	Kampar virus	2006	Human	Malaysia
PRV5HK	HK23629/07	2007	Human	Indonesia
PRV6XR	Xi River virus	2006	Bat (*Rousettus leschenaultii*)	China
PRV7S	Sikamat virus	2010	Human	Malaysia
PRV8B	HK46886/09	2009	Human	Indonesia
PRV9HK	HK50842/10	2010	Human	Indonesia
PRV10M	Miyazaki-Bali 2007	2007	Human	Indonesia
PRV11C	Cangyuan virus	2012	Bat (*Rousettus leschenaultii*)	China
PRV12I	Indonesia/2010	2010	Bat (*Pteropus vampyrus*)	Indonesia
PRV13P	Samal-24	2013	Bat (*Eonycteris spelaea*)	Philippines
PRV14P	Talikud-80	2013	Bat (*Rousettus amplexicaudatus*)	Philippines
PRV15G	Garut-69	2017	Bat (*Pteropus vampyrus*)	Indonesia
PRV16K*	Kasama virus	2017	Bat (*Lissonycteris angolensis ruwenzorii*)	Uganda

* identified in this study.

**Table 2:** Mantel test showing the correlation of geographic and genetic distances for PRV S1, S2, and S4.

PRV Genome Segment	Virus Protein(s)	Mantel’s r (rM)	*p*-Value (Two-Tailed) [99% CI]
S1	P10 (FAST), P17 (unknown), σC (cell attachment protein)	0.462	<0.0001 *
S2	σ1 (major inner-capsid protein)	0.229	0.031 [0.027–0.036] *
S3	σNS (nonstructural replication protein)	0.132	0.179 [0.169–0.189]
S4	σ2 (major outer-capsid protein)	0.373	<0.0001 *

* significant to α = 0.05.

## Supplementary Materials

The following are available online at https://www.mdpi.com/1999-4915/12/7/740/s1, Figure S1: Ka/Ks annotated tree of cell-attachment protein ORF of *Pteropine orthoreovirus* Segment 1, Table S1: Accession numbers and locations of PRVs used in this study.
