# Supplementary material for: Pteropine Orthoreovirus in an Angolan Soft-Furred Fruit Bat (Lissonycteris angolensis) in Uganda Dramatically Expands the Global Distribution of an Emerging Bat-Borne Respiratory Virus
Source: Viruses. 2020 Jul 9;12(7):740. doi: 10.3390/v12070740 (PMC7412351; doi:10.3390/v12070740)

Supplemental Figure 1:
Ka/Ks annotated tree of cell-attachment protein ORF of Pteropine orthoreovirus Segment 1

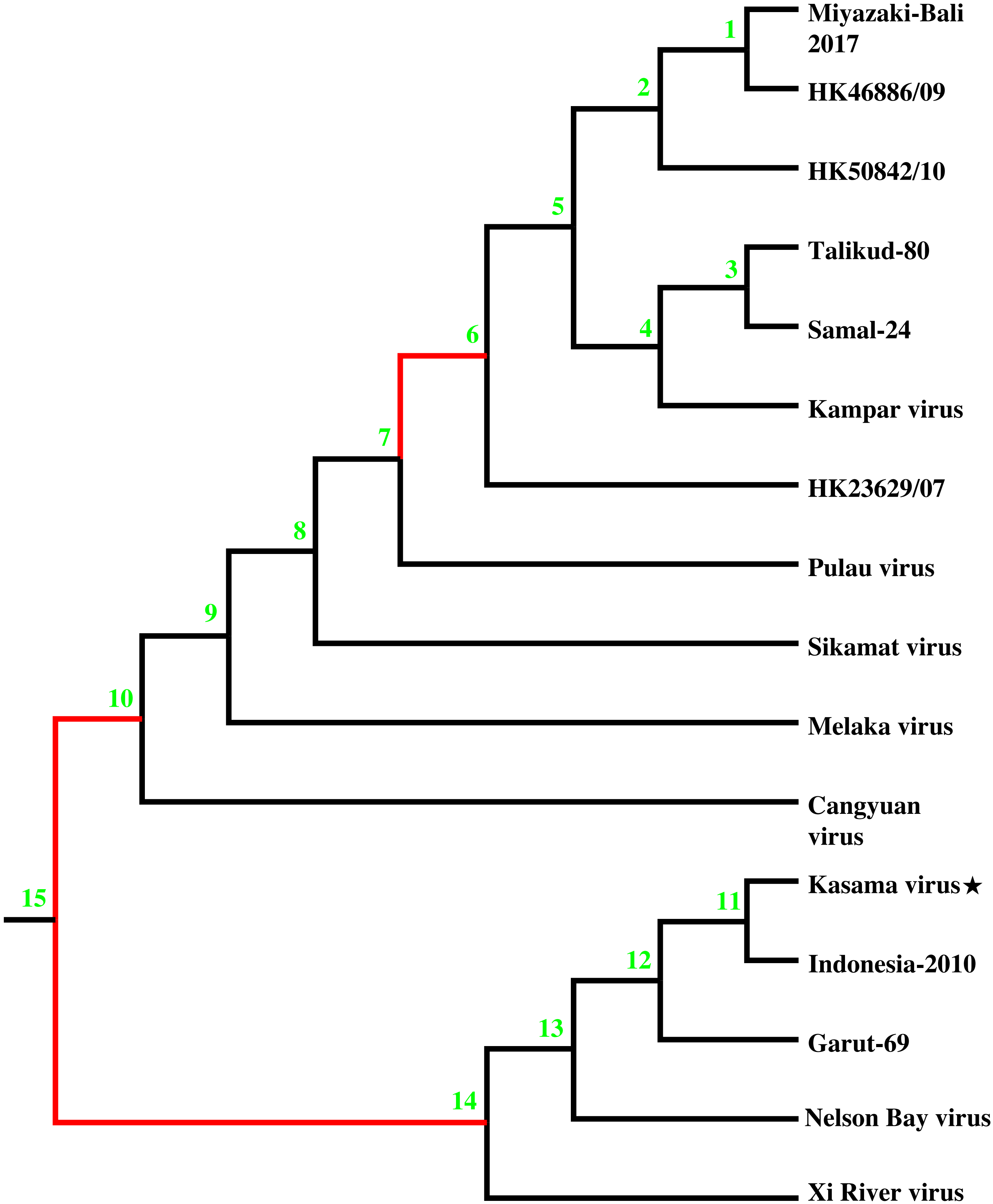

Supplement: Supplementary file 1 [file viruses-12-00740-s001.zip › Supplementals Revision/Supplemental Figure 1 REVISED.pdf]
